# An appraisal of how the vitamin A-redox hypothesis can maintain honesty of carotenoid-dependent signals

**DOI:** 10.1002/ece3.1364

**Published:** 2014-12-18

**Authors:** Mirre J P Simons, Ton G G Groothuis, Simon Verhulst

**Affiliations:** 1 Department of Animal and Plant Sciences, University of SheffieldSheffield, S102TN, UK; 2 Behavioural Biology, University of GroningenPO-Box 11103, 9700CC, Groningen, the Netherlands

**Keywords:** Birds, carotenoids, coloration, meta-analysis, sexual selection

## Abstract

The vitamin A-redox hypothesis provides an explanation for honest signaling of phenotypic quality by carotenoid-dependent traits. A key aspect of the vitamin A-redox hypothesis, applicable to both yellow and red coloration, is the hypothesized negative feedback of tightly regulated Vitamin A plasma levels on the enzyme responsible for sequestering both Vitamin A and carotenoids from the gut. We performed a meta-analysis and find that vitamin A levels are positively related to carotenoid plasma levels (*r *=* *0.50, *P* = 0.0002). On the basis of this finding and further theoretical considerations, we propose that the vitamin A-redox hypothesis is unlikely to explain carotenoid-dependent honest signaling.

## Introduction

Carotenoid-dependent traits are found throughout the animal kingdom and are especially ubiquitous in birds. The color intensity of these traits is presumed to honestly signal phenotypic quality (Alonso-Álvarez and Galván 2011; Simons et al. 2012a), and female choice for these traits has been demonstrated (e.g., Simons and Verhulst 2011; Toomey and McGraw 2012). Carotenoid-dependent traits have been hypothesized to signal oxidative stress state and immunocompetence, because of carotenoids' alleged antioxidant (von Schantz et al. 1999) and immune-enhancing properties (Lozano 1994). There is evidence for both these hypotheses, yet effect sizes are low, suggesting that there could be additional honesty maintaining mechanisms operating (Svensson and Wong 2011; Simons et al. 2012b). The Vitamin A-redox hypothesis provides such an alternative mechanism (Hill and Johnson 2012).

The vitamin A-redox hypothesis is based on the constraints imposed by vitamin A regulation and its shared biochemical pathways with carotenoids. Hill and Johnson built upon the physiological actions of vitamin A, which are deeply rooted into physiology, and hypothesize that these physiological actions might therefore maintain honesty of carotenoid-dependent signals. For instance, vitamin A regulates intracellular signaling involved in for example early development and B lymphocyte activation. Vitamin A does so by acting as a transcriptional activator across the genome. More specifically, all-trans retinoic acid, a product derived from vitamin A, drives transcription in a redox-dependent manner. Hill and Johnson argued that carotenoid uptake and metabolism are impaired when vitamin A homeostasis is compromised, rendering carotenoid-dependent traits honest signals of condition. The thorough biochemical background of vitamin A, carotenoids and their connections outlined by Hill & Johnson provide exciting new avenues for research, integrating biochemistry into behavioral ecology.

**Figure 1:**
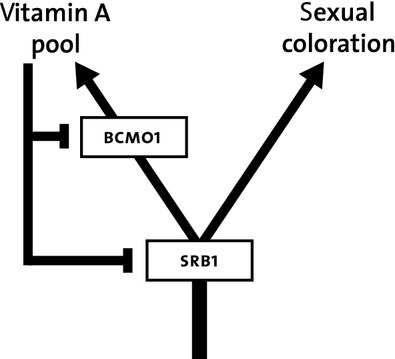
The negative feedback of vitamin A levels on carotenoid uptake via retinoid acid according to Hill and Johnson (2012). SR-B1 takes up carotenoids (pro-vitamin A carotenoids *and* carotenoids) and vitamin A from the gut and BCMO1 convert pro-vitamin A carotenoids to vitamin A. Negative feedback from the vitamin A pool regulated by retinoid acid regulates uptake from the gut to maintain vitamin A homeostasis.

We explored whether the vitamin A-redox hypothesis can explain carotenoid-dependent signal honesty by focusing on one key aspect of the hypothesis, negative feedback of vitamin A levels on carotenoid uptake, which is the proposed mechanism by which disturbances in vitamin A homeostasis can disrupt carotenoid uptake. This key biochemical constraint assumed in the vitamin A-redox hypothesis (Hill and Johnson 2012), that is applicable to *both* yellow and red carotenoid-dependent coloration, is that pro-vitamin A carotenoids and, importantly, also *other* carotenoids (more commonly used in coloration than pro-vitamin A carotenoids) are taken up by the same protein in the gut, SR-B1, and that BCMO1 converts pro-vitamin A carotenoids to vitamin A. Vitamin A homeostasis is (in part) regulated by negative feedback of retinoid acid on BCMO1 and SR-B1. Hill and Johnson hypothesized that this negative feedback links the vitamin A pool and thus vitamin A homeostasis, to carotenoid uptake and availability for its use in trait pigmentation (Fig.1), and thereby ensures signal honesty. Note that knowledge of these feedbacks is largely based on research in mammals, and were assumed by Hill and Johnson to be similar in birds because they are supposedly evolutionary conserved. However, whether these feedback mechanisms do indeed operate similarly in birds and mammals remain to be verified. With this caveat in mind, we can however evaluate aspects of the vitamin A-redox hypothesis. Because of the presumed negative feedback (Fig.1) of vitamin A levels on carotenoid uptake, vitamin A levels may be either (1) negatively related with carotenoid levels if negative feedback is sufficiently strong as assumed in the vitamin A-redox hypothesis, or it may (2) decouple carotenoid levels from vitamin A levels, because vitamin A homeostasis is maintained and the carotenoid pool reflects perturbations in vitamin A homeostasis via negative feedback on carotenoid uptake or (3) If negative feedback is not sufficiently strong, vitamin A and carotenoid may be positively related if they covary in the diet or for other physiological reasons not related to sexual signaling. The latter finding would falsify a key aspect of the Vitamin A-redox hypothesis, in that the postulated processes may still occur, but are apparently overridden by other processes making their net effect negligible in the context of honest signaling. Such processes could for example be between-individual variation in quality resulting in differences in the ability to maintain vitamin A homeostasis or the ability to bear the handicap of reduced vitamin A homeostasis – also generating a positive relationship between vitamin A, carotenoids and associated sexual signaling. In humans in which SBR1, BCMO1 negative feedback has been well confirmed, a positive correlation between retinol and carotenoids is still apparent (Russell-Briefel et al. 1985; Schunemann et al. 2001), suggesting that between-individual variation in retinol and carotenoid levels is more important than the negative feedback via BCMO1 and SRB1.

## Methods

To distinguish between these three options, we gathered all available data on the relationship between vitamin A (specifically retinol) and carotenoid plasma levels in adult birds and subjected these to a meta-analysis. We searched the literature using Google Scholar with the following search terms (with the last search in August 2014): vitamin A, retinol, carotenoids, birds, and contacted authors of the eligible papers if the correlation was not directly reported, but vitamin A and carotenoid levels were reported. We retrieved observational data from six species of birds (Blount et al. 2003; Hõrak et al. 2004; Larcombe et al. 2008; Arnold et al. 2010; Martinez-Haro et al. 2011), and with the exception of two studies (Hõrak et al. 2004; Martinez-Haro et al. 2011), that were conducted on captive birds. From three additional eligible publications, we failed to obtained the data (Møller et al. 2005; Navarro et al. 2010; Giraudeau and McGraw 2014). All studies employed high-performance liquid chromatography (HPLC) as measurement technique, ruling out cross-reactivity causing spurious correlations. We could however not distinguish between types of carotenoids and although this should generally be preferred (McGraw 2006), total carotenoid plasma content correlates strongly to carotenoid-dependent signal expression across species (Simons et al. 2012b). Random-effect meta-analyses were performed using *metafor* (Viechtbauer 2010) in R (R Development Core Team 2011).

## Results

**Figure 2:**
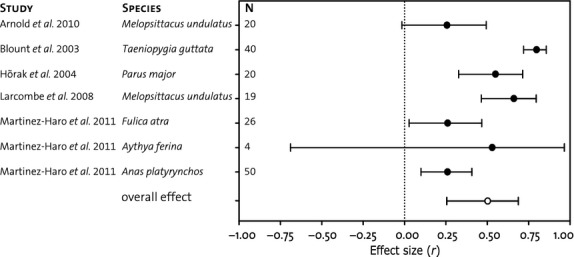
Correlations (closed dots) between retinol and total carotenoids in plasma with the corresponding 95% confidence intervals. The overall effect as estimated by random-effect meta-analysis, and the corresponding 95% confidence interval is depicted with an open dot.

All studies showed a positive correlation between carotenoids and retinol, which was overall highly significant (*r *=* *0.50, *P* = 0.0002; Fig.2; without apparent publication bias: rank test, *P* = 0.36 and funnel plot inspection). This suggests that the negative feedback of vitamin A levels on carotenoid uptake is not sufficiently strong to constrain carotenoid uptake in order to render carotenoid-dependent signal honest. Although the sample size of the meta-analysis is relatively small, patterns are consistent across studies (Fig.2), increasing our confidence in the overall estimate. Note also that whether the species exhibited a carotenoid-dependent trait as an adult (*Taeniopygia guttata, Parus major* and *Anas platyrynchos*) or not did not affect the overall correlation when tested as a moderator (*Q* = 0.49, *P* = 0.48). First, pooling across *Melopsittacus undulates* across the two separate studies included also did not change any of the conclusions.

## Discussion

Our meta-analysis, although relatively low on sample size, but showing a very consistent overall effect, is to our best knowledge the first test of a key aspect of the vitamin A-redox hypothesis. Because this hypothesis currently lacks any in vivo support in birds, our findings challenge the validity of the vitamin A-redox hypothesis in general, warranting a further critical review of this hypothesis. In addition to our empirical finding, there are also theoretical reasons to doubt the explanatory power of the vitamin A-redox hypothesis. In general, it is debatable whether shared biochemical pathways can maintain honesty by providing a physiological evolutionary constraint (Arnold 1992) in the face of sufficiently strong sexual selection. A biochemical constraint becomes unlikely if it can be overcome by stepwise pathway evolution. Here, we explore qualitatively, possible mutations that allow cheating of the constraint mechanism proposed by Hill and Johnson, if it were to operate. If a mutation would arise that causes SR-B1 to be less sensitive to retinoid acid, it will increase sexual coloration inducing the cost of a decrease in vitamin A homeostasis, because the balance in negative feedback between BCMO1 and SR-B1 is altered. This mutant is now outcompeting its rivals with superior sexual coloration, acting as a handicap signal of vitamin A homeostasis. Another mutation can however restore this balance via a mutation to increase sensitivity of BCMO1 to negative feedback via retinoid acid. Such a cycle can continue until the degree of negative feedback on BCMO1 and SR-B1 is balanced such that uptake of carotenoids can continue without direct dependence upon vitamin A levels. BCMO1 has the potential to effectively regulate retinol levels via cleavage of pro-vitamin A carotenoids without the need for affecting carotenoid uptake. Such mutations are not merely hypothetical given that polymorphisms in the BCMO1 gene in humans strongly influence levels of *β*-carotene and conversion to retinol (Leung et al. 2009).

Another consideration that sheds doubt on the vitamin A-redox hypothesis is that additional regulatory pathways are likely to exist, given that the proportion of nonvitamin-A precursor carotenoids and pro-vitamin A carotenoids will not be constant in the diet and only the latter affects the retinol pool. This provides another reason why regulation at the uptake level, limiting carotenoid acquisition, is unlikely to be constrained by vitamin A homeostasis. Moreover, short-term dynamics depleting vitamin A stores – a detrimental event reasoning from the crucial role of vitamin A in physiological processes – will actually lead to a strong upregulation of *both* vitamin A and carotenoid uptake, thereby potentially increasing carotenoid-dependent coloration after a detrimental life-event, in striking contrast with the hypothesized role of carotenoid-dependent signals as an indicator signal of phenotypic quality. Different mechanisms are also plausible. For example, beta-carotene dioxygenase 2 (BCDO2) removes harmful oxidized carotenoids from mitochondria (Johnson and Hill 2013) and regulates carotenoid-based integument coloration of the domestic chicken (Eriksson et al. 2008). This may suggest different windows in which carotenoids are beneficial and harmful thereby possibly generating signal honesty (Johnson and Hill 2013) and explaining context-dependent effects of carotenoid supplementation on reproduction (Simons et al. 2014a).

These considerations, together with the meta-analysis on the relationship between carotenoids and vitamin A, suggest that the joint pathways of uptake of vitamin A and carotenoids, as postulated in the vitamin A-redox hypothesis, are unlikely to be strong enough to maintain signal honesty. Resource allocation and acquisition of carotenoids or alternative hypotheses (Hartley and Kennedy 2004; Svensson and Wong 2011; Simons et al. 2012b, 2014b) are therefore more likely to maintain honesty in our view. This also encompasses pro-vitamin A carotenoids allocation away from ornamentation toward serving as retinoic acid resource (Hartley and Kennedy 2004). Alternatively, between-individual variation in vitamin A levels and the associated carotenoid levels, resulting in differential expression of sexual coloration, could reflect differences between individuals in the extent to which they tolerate possible costs of reduced vitamin A homeostasis. Thus, individuals that acquire higher amounts of carotenoids, and hence are more ornamented, may be tolerating higher costs and may thus hence be of higher quality. This would mean that such individuals reduce negative feedback of vitamin A on shared vitamin A and carotenoid uptake to increase carotenoid levels, which may impose a cost in terms of reduced vitamin A homeostasis or higher vitamin A levels in general. In particular, variability in vitamin A levels could impose a cost, given that it may be possible to reduce the sensitivity of vitamin A-dependent processes negating possible costs of higher vitamin A levels, but variability in vitamin A is likely to result in possibly detrimental variability in these vitamin A-dependent physiological processes. This latter hypothesis can be tested by examining within-individual variability in vitamin A levels (homeostasis) and relating this to carotenoid uptake or levels, and sexual coloration.

In general, it will be experimental studies that can demonstrate a causal link between vitamin A, carotenoids, and carotenoid-dependent sexual signaling. Moreover, the hypothesized constraints might not be detectable in healthy unchallenged individuals and more insight could be gained by evaluating vitamin A homeostasis and connections to signaling in a stressful environment. Note any effect dependent on such an environment will have relatively low signaling value because it will not differentiate amongst healthy individuals, whereas choice for carotenoid-dependent signals can be strong in unchallenging environments (Simons and Verhulst 2011). In conclusion, our meta-analysis and subsequent considerations do not support a dominant role for vitamin A regulation in maintaining honesty of carotenoid-dependent signals, but we acknowledge that experimental tests are required for a more conclusive statement on the explanatory power of the vitamin A-redox hypothesis.
